# The Use of the Adaptation Potential Reduction Model for Reproductive Toxicity Research In Vivo

**DOI:** 10.1155/2020/8834630

**Published:** 2020-11-21

**Authors:** Nadezhda V. Tyshko, Elvira O. Sadykova, Svetlana I. Shestakova, Nikolay S. Nikitin, Marina D. Trebukh, Maria S. Loginova, Valentina A. Pashorina, Valentin M. Zhminchenko

**Affiliations:** Federal State Budgetary Scientific Institution, Federal Research Centre of Nutrition, Biotechnology and Food Safety, Moscow, Russia

## Abstract

The modeling of adaptation potential decrease in rats due to modification of the diet's vitamin–mineral composition allows to increase animals' sensitivity to toxic load in reprotoxicological experiments. The threshold values of vitamins B1, B2, B3, and B6 and mineral substances Fe^3+^ and Mg^2+^ in the diet, which lead to a considerable reduction of laboratory animals' adaptation potential, have been determined as 19% (from the basic level in the diet) for males and 18% for females. The efficiency of this model has been confirmed in a reprotoxicological experiment with glyphosate as a toxic factor: the action of the toxic factor against the background of reduced availability of B vitamins and salts Fe^3+^ and Mg^2+^ led to significant changes in such indicators of reproductive function as mating efficiency, postimplantation loss, and the total number of alive pups, while the toxic effect of glyphosate was not so pronounced against the normal level of essential substances. The obtained results prove that this adaptation potential reduction model can be recommended for the research of the low-toxicity objects reproductive toxicity in rats and for the safety assessment of novel food, in particular.

## 1. Introduction

In the safety assessment of a novel food and genetically engineered organisms of plant origin, in particular, the central place is occupied by reprotoxicological studies to prove the absence of remote negative effects that may manifest themselves only in the next generation. The complex research of reproductive function, pre- and postnatal progeny development usually involves the study of a large number of parameters, with a wide range of physiological fluctuations in each, whereas the heterogeneous distribution of some indicators' values complicates the interpretation of the results, especially under conditions of toxic impact of low intensity [1–3].

Indeed, the recognition of an organism's response in the range of physiological adaptation/pseudoadaptation (compensated latent pathological process) [4] is a very difficult task even for a modern laboratory. For this reason, the simulation of additional load, which reduces the adaptation potential and, consequently, eliminates the possibility of compensation for the pathological process, is a promising tool to improve the diagnostic validity of the experiment results.

Modeling of additional load that reduces the adaptation potential and, consequently, excludes the possibility of compensatory pseudoadaptation is a promising tool to improve the diagnostic reliability of the experiment results [1–4]. Since one of the simplest and most effective ways to reduce the adaptive potential of laboratory animals' organisms is to modify the diet composition, several series of studies were carried out. The result of these studies was the creation and efficiency confirmation of the *in vivo* model, which increases the susceptibility of the rats to the toxic factors action by reducing the supply of B vitamins (B1, B2, B3, and B6) and minerals (Fe^3+^ and Mg^2+^) [1–3]. The threshold values of these essential substances in the diet, which lead to a considerable reduction of laboratory animals' adaptation potential, have been determined as 19% (from basic levels of AIN93 diet) for males and 18% for females. This modification of diet composition was recommended for use as a model to increase the sensitivity of rats to toxic factors in the study of low-toxicity objects [1–4].

This work was aimed at proving the efficiency of adaptation potential reduction model of rats (against the background of the known toxic factor) for application in reprotoxicological experiments.

Herbicide glyphosate was used as a toxic factor and now is one of the most widely used herbicides in the world, whose toxic effect is well studied [5]. Glyphosate (N-(phosphonomethyl)glycine) is a nonselective herbicide; its mechanism of action is based on blocking the synthesis of some essential aromatic amino acids through affecting the metabolism of shikimic acid. The key stage of this process is the synthesis of 5-enolpyruvylshikimate-3-phosphate from phosphoenolpyruvate and shikimate-3-phosphate catalyzed by 5-enolpyruvylshikimate-3-phosphate synthase. This particular enzyme is the target of glyphosate action. The described type of shikimic acid metabolism is typical of plants, algae, bacteria, fungi, and protozoans; other living forms including insects, fish, birds, mammals, and humans do not have such a metabolic pathway [6, 7].

Glyphosate is characterized by a low capacity for cumulation and can be excreted with feces and urine in a virtually unchanged form when enters the body [8]. Approximately 3% of introduced glyphosate is transformed into aminomethylphosphonic acid, the toxic effect of which is several times stronger than the glyphosate itself [9–11]. Thus, we have solid grounds to believe that the toxicant chosen will make an impact on laboratory animals.

## 2. Materials and Methods

### 2.1. Animals and Experiment Design

The experiment of total duration of 155 days was conducted on Wistar rats of generation F0 (176 females and 64 males) and F1 (684 pups and 265 fetuses). The animals of parental generation F0 (initial age ∼30 days) were divided equally and randomly into four groups: two control and two test groups, 16 males and 44 females in each. Rats of the control groups received semisynthetic casein diets [12, 13] with two different contents of vitamins B1, B2, B3, and B6 and minerals Fe^3+^ and Mg^2+^: the 19% content group—“Control-19” and the 75% content group—“Control-75.”

Modifications within the AIN-93 [12, 13] diet included a decrease in the content of B vitamins (thiamine, riboflavin, niacin, and pyridoxine) and iron and magnesium salts in the salt and vitamin mixtures (Table 1). Feed and water access ad libitum.

Glyphosate herbicide was chosen as a toxic factor, whose effect on reproductive function is described in detail [14–16]. Based on the toxicological data of dose/exposure dependence at oral intake [14–16], there was used a certainly acting dose of glyphosate 2.5 mg/kg of body weight daily during the whole period of the experiment. The animals of the test groups received glyphosate with the similar diets: “Test-19” and “Test-75,” respectively.

### 2.2. Reproduction and Developmental Assessment

Reproductive function was estimated by the fertility of animals F0 and by the character of prenatal and postnatal development of F1 progenies. The work with animals and all procedures performed were done according to the American Veterinary Medical Association (AVMA Guidelines for the Euthanasia of Animals: 2013 Edition) [17] and Rules of laboratory practice approved by Order of the Ministry of Health of the Russian Federation No. 193n of 01/01/2016.

The euthanasia procedure was completed after the animals were deprived of food for 12 h via physical method (decapitation).

Fertility was determined by (1) the mating efficiency as a ratio of fertilizing ability of males over the total number of cohoused males, or as a ratio of pregnant females over the total number of cohoused females, and (2) endocrine function of the ovaries (content of estradiol, progesterone, and testosterone in the pregnant females' blood serum).

For prenatal development assessment, F0 females were euthanized on the 20th day of pregnancy (one day prior to the expected day of delivery). The uteruses were removed by cesarean section and the uteruses and the fetuses were examined. The females were examined macroscopically for any structural abnormalities or pathological changes. The number of ovarian corpora lutea, resorptions, implantation sites, the number of alive and dead fetuses, and the preimplantation loss (i.e., difference between the number of ovarian corpora lutea in ovaries and the number of implantation sites in uterus) as well as the postimplantation loss (i.e., difference between the number of implantation sites in uterus and the number of alive fetuses) were determined. The fetuses were extracted, examined macroscopically; weight and craniocaudal size were determined and examined by Wilson's method and Dawson's method [18–20].

Postnatal F1 progeny development was being assessed during the first month of life by counting the number of alive and dead pups, dynamics of body weight and length, and physical developmental landmarks (i.e., ear unfolding, first coat, incisor eruption, eye opening, testicle lowering, and vagina opening). Pups' body weight and length were measured on postnatal days 2, 5, 10, 15, 20, and 25 [20]. The average litter size, the male-to-female ratio, and the postnatal survival index were calculated from the 1st to the 25th day of life (i.e., the ratio between the number of pups being alive on the 25th day and the total number of pups born alive). This assessment was performed in accordance with the Russian Guidelines [21, 22] and was also considering the OECD methodical guidelines [23].

The rats had been kept in plastic cages with wood shavings, in ventilated and heated (T ∼20–23°C) room with natural light. All animals were observed once daily for mortality, moribundity, and overt signs of toxicity. Individual body weights of F0 animals were obtained once weekly for 30–100 days of age [20].

### 2.3. Statistical Analysis

Statistical analysis was executed with the use of SPSS 17.0 software package (IBM, USA). Reliability assessment of the mean figures differences of data normally distributed was analyzed by one-way ANOVA. Statistical significance was assigned at the *p* < 0.05 level [24]. The data were presented as *M* ± SE and min-max, where *M* was the mean, SE was the standard error, and min-max were the minimal and maximal values, as well as percentage or absolute figures.

## 3. Results and Discussion

**Figure 1:**
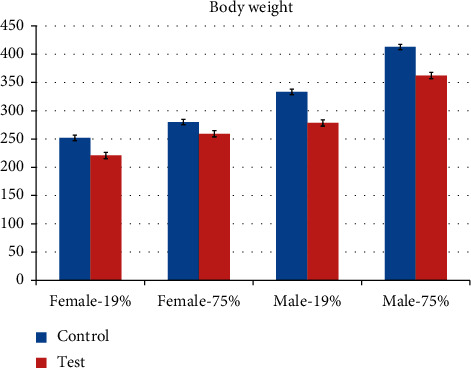
The body weights of rats F0 on the 100th day of life.

The general condition of all animals was satisfactory. By the 100th day of life corresponding to the age of physiological maturity optimal for mating, the statistically significant differences in body weights of the experimental groups have been observed: the control groups showed a body weight increase in the row Control-19 < Control-75 at 252.1 ± 3.1 g and 280.1 ± 4.8 g in females, 333.3 ± 6.9 g and 412.8 ± 9.9 g in males, respectively. In test groups, there was a similar trend (Test-19 < Test-75), but the body weights were slightly lower than in corresponding control groups (221.0 ± 2.5 g and 259.3 ± 2.8 g in females and 278.4 ± 5.5 g and 362.4 ± 5.5 g in males, resp.). The differences in body weights of Control-75 and Test-75 groups stand at 7% in females (*p* < 0.05), 12% in males (*p* < 0.05), and those of Control-19 and Test-19 groups stand at 12% in females and 17% in males (*p* < 0.05), respectively (Figure 1).

**Figure 2:**
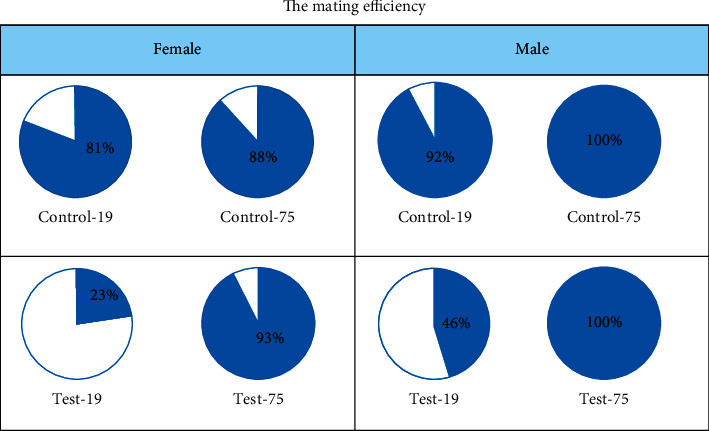
The mating efficiency of rats F0.

The mating efficiency of control group for females was 81% in Control-19 and 88% in Control-75; for males—92% in Control-19 and 100% in Control-75; the mating efficiency of test group females was 23% in Test-19 and 93% in Test-75; for males—46% in Test-19 and 100% in Test-75 (Figure 2). Evaluation of F0 rats generative function revealed a certain correlation between the effects of glyphosate and essential substances levels: there were no differences between Control-75 and Test-75 groups (mating efficiency corresponded to the typical average values of rats), while Control-19 and Test-19 groups demonstrated a significant differences in mating efficiency: the females from test group had this indicator 58% lower than in control, the males from test group—46% lower.

Throughout the pregnancy, no adverse effects on females behavior and appearance were observed; the relative body weight gain of Test-75 females was slightly lower than that of Control-75 females; in groups with low supplying of essential substances, the relative body weight gain was much lower than that of females with optimal provision, but the differences between Control-19 and Test-19 groups were less expressed.

The survey macroscopic examination of pregnant females' internal organs did not reveal any pathological changes in all groups; the weights of internal organs were within the limits of physiological fluctuations. The study of gonads' endocrine function on the 20th day of pregnancy did not show any differences between the groups; the contents of sex hormones were within the normal range.

Analysis of F1 fetuses prenatal development found that the number of ovarian corpora lutea and implantation sites in the females of control and test groups did not demonstrate significant differences; the values of these indicators fell within the limits of physiological fluctuations (Table 2). The average number of alive fetuses in rats of Test-75 group was 17% (*p* > 0.05) lower than in Control-75 group but did not exceed the norm, while the differences between Test-19 and Control-19 groups were 72% (*p* < 0.05). The decrease in the number of alive fetuses in Control-19 group reached 32% (*p* > 0.05) compared to Control-75 group.

Preimplantation loss in both test groups was similarly above the norm, but showed some differences between control groups: in Control-19, this indicator was slightly above the norm and 56% (*p* > 0.05) higher against Control-75. Postimplantation loss in groups Control-75 and Test-75 reached the upper limit of the norm and had no significant differences; the average values of this indicator in Control-19 rats were 151% (*p* > 0.05) higher against Control-75 group and 155% (*p* < 0.05) higher in Test-19 rats against Control-19 group.

The fetuses' zoometric indices and the internal organs weights varied within the physiological norm; Test-75 fetuses expressed no reliable differences from Control-75. Test-19 fetuses on the whole did not differ from Control-19, but some distinctions were noted in craniocaudal size, an absolute and relative weight of kidneys: these indices in Test-19 group were slightly lower than in Control-19 group—by 5%, 21%, and 20% (*p* < 0.05), respectively.

Postnatal development of Test-75 pups (total number of pups, average litter value) did not differ from Control-75 group, while Test-19 indices were 95% and 35% lower against Control-19 group, respectively (Table 3).

The analysis of physical development of F1 pups did not reveal any deviations from the norm. The survival rate during the first month of life in Control-75 and Test-75 groups was 98% and 99%. According to the scientific data, the Wistar rats are characterized by the relative variability of certain reproductive function indicators; hence, 98-99% value corresponded to the highest level of survival rate for rats of this line.

In the Control-19 group, the survival rate was 39%, which is much lower than the typical values for this line of animals. In Test-19 group, the survival rate stood at 100%, with all 9 pups survived, although such a limited sample cannot be considered representative, which does not allow us to assess the result.

The effects observed could be brought about by a general decline of the adaptive potential, as well as the shortage of essential substances affecting reproductive function. Vitamins are crucial to maintain gametogenesis, participate in redox processes and many enzymatic reactions [25]. Thiamine (B1) deficiency leads to a higher embryonic mortality and pathological changes in the internal organs of the fetuses due to cardiovascular and nervous system development disorders in the implantation period of blastocyst and gastrula [26]. Deficiency of riboflavin (B2) tends to slow down the growth and differentiation of the fetal tissues and leads to missed miscarriage in severe cases [27, 28]. Niacin (B6) is involved in the synthesis of steroid, including sex hormones, and hormonal status disorders caused by its deficiency result in fertility decline and pregnancy termination [29]. Pyridoxine (B12) deficiency brings about anorexia, weight loss, pathological changes of internal organs (liver, lungs, and ovaries), slower puberty, and sterility [27]. Deficiency of fat-soluble vitamins can also affect a reproductive function: given retinol deficiency, the number of spermatogonia and sperm cells, as well as a testosterone synthesis and a number of fetuses' ovarian cells tend to decrease, while the above deficiency was found to increase the content of follicle stimulating hormone, atypical nuclei in Sertoli cells, germ cell apoptosis [30, 31]. Tocopherol involved in the redox processes of spermatogenesis prevents lipid peroxidation in sperm cells [32]; tocopherol deficiency reduces sperm motility and leads to depression of reproductive function.

Many enzymes are characterized by the specificity of the active centers formed by their component metals including copper, zinc, manganese, selenium, and gem iron. Therefore, the intake shortage may result in a decrease of the corresponding enzymes activity. Deficiency of trace elements in mammalian diets leads to abnormalities of gametogenesis and endocrine function of gonads, reduced fertility, teratogenic effects, and changes in pregnancy duration. Deficiency of calcium, iron, manganese, and copper in diets appears to cause premature labor, anemia, skeleton ossification disorders, and central nervous system disorders [33–35].

The iron content in the body is regulated mainly by its absorption level in the intestines. Considering that hemoglobin accounts for 60–70% of all iron content, the iron deficiency in the diet shall be primarily regarded as a threat of anemia [36]. Iron deficiency (7 mg/kg) in the diet deteriorates the milk quality in lactating females: on the 21st day of lactation, offspring demonstrated a delay in growth and hemoglobin level decrease in blood [34]. Magnesium deficiency of 30 mg/kg in the diet has an adverse effect on spermatogenesis, reducing the generative function. In females, a prolonged magnesium deficiency leads to miscarriages, infertility, increased number of resorptions, and during lactation—to the reduction of pups' body weight [33].

Thus, the reduced content of B vitamins and salts Fe^3+^ and Mg^2+^ in the semisynthetic casein diet of laboratory animals provides a well-detectable and unambiguously interpreted response of the reproductive system to toxic factors due to the complex action of essential substances deficit on the reproductive function and adaptive potential of the body.

## 4. Conclusions

The action of the toxic factor against the background of reduced availability of B vitamins and salts Fe^3+^ and Mg^2+^ led to significant changes in such indicators of reproductive function as mating efficiency, postimplantation loss, and the total number of alive pups, while the toxic effect of glyphosate was not so pronounced against the normal level of essential substances. The obtained results prove that this adaptation potential reduction model can be recommended for the research of the low-toxicity objects reproductive toxicity in rats and for the safety assessment of novel food, in particular.

## Figures and Tables

**Table 1:** Diet modifications.

Ingredient	Groups, % of essential substances supplying (as initial diet AIN-93 was used [13])	
	75%	19%
Vitamins, g/kg of vitamin mixture		
Thiamine (B1)	3	0.76
Riboflavin (B2)	2.25	0.57
Niacin (B3)	11.3	2.85
Pyridoxine (B6)	3.75	0.95

Mineral substance, g/kg of salt mixture		
Magnesium oxide	18	4.56
Iron citric acid	4.545	1.1514

**Table 2:** Prenatal development of progeny F1.

Indicators		Group				Historical control data
		Control		Test		
		Control-19	Control-75	Test-19	Test-75	
Number of pregnant females		8	7	5	9	—

Number of ovarian corpora-lutea	Total	119	112	65	132	5–23
	*M* ± *m*	14.88 ± 0.67	16.00 ± 0.82	13.00 ± 1.95	14.67 ± 0.82	
	*min–max*	*13–19*	*13–19*	*6–18*	*10–18*	

Number of implantation sites	Total	100	101	53	101	3–18
	*M* ± *m*	12.50 ± 0.73	14.43 ± 0.81	10.60 ± 2.52	11.22 ± 1.75	
	*min–max*	*9–15*	*11–17*	*2–17*	*2–17*	

Number of alive fetuses	Total	69	89	12	95	2–18
	*M* ± *m*	8.63 ± 1.36	12.71 ± 0.97	2.4 ± 1.69^*∗*^	10.56 ± 1.72	
	*min–max*	*1–14*	*7–14*	*0–9*	*1–16*	

Number of resorptions	Total	30	12	41	6	0–10
	*M* ± *m*	3.75 ± 1.36	1.71 ± 0.61	8.20 ± 2.85	0.67 ± 0.37	
	*min–max*	*0–12*	*0–4*	*1–15*	*0–3*	
Number of dead fetuses		2	0	1	0	—

Preimplantation loss						
%	*M* ± *m*	15.23 ± 5.39	9.74 ± 2.72	24.77 ± 11.74	24.11 ± 11.21	15 ± 1
	*min–max*	*0–37*	*0–22*	*0–67*	*0–83*	*0–80*

Absolute value	Total	19	11	12	31	2.2 ± 0.2
	*M* ± *m*	2.38 ± 0.91	1.57 ± 0.48	2.40 ± 0.81	3.44 ± 1.68	
	*min-max*	*0–7*	*0–4*	*0–4*	*0–15*	*0–13*

Postimplantation loss						
%	*M* ± *m*	30.64 ± 10.41	12.21 ± 4.87	78.22 ± 17.20^*∗*^	9.37 ± 5.65	7 ± 1
	*min–max*	*0–93*	*0–36*	*10–100*	*0–50*	*0–100*

Absolute value	Total	32	12	41	6	0.8 ± 0.1
	*M* ± *m*	4.00 ± 1.46	1.71 ± 0.61	8.20 ± 2.85	0.67 ± 0.37	
	*min–max*	*0–13*	*0–4*	*1–15*	*0–3*	*0–10*

^*∗*^Significant differences compared to the appropriate control (*p* < 0.05, ANOVA, Student's *t*-test).

**Table 3:** Postnatal development of progeny F1.

Indicators		Group			
		Control		Test	
		Control-19	Control-75	Test-19	Test-75
Number of pregnant females		21	23	8^*a*^	24
Number of females giving birth to a litter		21	23	6	24
Number of litters killed by mothers on the 1st day of life		0	0	4	1
Total number of pups		168	252	9	255
Of those—stillborn		17	3	0^*b*^	7
Mean litter size	*M* ± *m*	6.88 ± 1.64	10.83 ± 0.87	4.50 ± 2.50	10.78 ± 0.63
	*min–max*	2–14	3–19	2–7	3–16

^*a*^In 2 females, the pregnancy was diagnosed based on the dynamics of body weight (increase of more than 70 g in 20 days), facts of delivery were not registered, and there were no pups in the cages (probably killed by mothers after birth). ^*b*^It is impossible to estimate since the facts of delivery were not registered.

## Data Availability

The data used to support the findings of the present study are available from the corresponding author upon request.
